# Fortifying Butterfat with Soybean Oil Attenuates the Onset of Diet-Induced Non-Alcoholic Steatohepatitis and Glucose Intolerance

**DOI:** 10.3390/nu13030959

**Published:** 2021-03-16

**Authors:** Victor Sánchez, Annette Brandt, Cheng Jun Jin, Dragana Rajcic, Anna Janina Engstler, Finn Jung, Anika Nier, Anja Baumann, Ina Bergheim

**Affiliations:** 1 Department of Nutritional Sciences, R.F. Molecular Nutritional Science, University of Vienna, Althanstraße 14/UZAII, A-1090 Vienna, Austria; Victor.sanchez@univie.ac.at (V.S.); annette.brandt@univie.ac.at (A.B.); dragana.rajcic@univie.ac.at (D.R.); j.engstler@googlemail.com (A.J.E.); finn.jung@univie.ac.at (F.J.); anika.nier@univie.ac.at (A.N.); anja.baumann@univie.ac.at (A.B.); 2 Institute of Nutrition, SD Model Systems of Molecular Nutrition, Friedrich-Schiller University of Jena, Dornburger Straße 25-29, 07743 Jena, Germany; taiji-2002@hotmail.com

**Keywords:** soybean oil, fatty liver, insulin resistance, endotoxin, PUFA

## Abstract

The addition of plant oils such as soybean oil (S) to a diet rich in saturated fatty acids is discussed as a possible route to prevent or diminish the development of metabolic disease. Here, we assessed whether a butterfat-rich diet fortified with S affects the development of early non-alcoholic steatohepatitis (NASH) and glucose intolerance. Female C57BL/6J mice were fed a standard-control diet (C); a fat-, fructose-, and cholesterol-rich diet (FFC, 25E% butterfat, 50% (wt./wt.) fructose, 0.16% (wt./wt.) cholesterol); or FFC supplemented with S (FFC + S, 21E% butterfat + 4E% S) for 13 weeks. Indicators of liver damage, inflammation, intestinal barrier function, and glucose metabolism were measured. Lipopolysaccharide (LPS)-challenged J774A.1 cells were incubated with linolenic and linoleic acids (ratio 1:7.1, equivalent to S). The development of early NASH and glucose intolerance was significantly attenuated in FFC + S–fed mice compared to FFC-fed mice associated with lower hepatic *toll-like receptor*-*4* mRNA expression, while markers of intestinal barrier function were significantly higher than in C-fed mice. Linolenic and linoleic acid significantly attenuated LPS-induced formation of reactive nitrogen species and *interleukin-1 beta* mRNA expression in J774A.1 cells. Our results indicate that fortifying butterfat with S may attenuate the development of NASH and glucose intolerance in mice.

## 1. Introduction

Insulin resistance has been shown to be a key factor in the development of metabolic diseases including non-alcoholic fatty liver disease (NAFLD) [1]. Indeed, the latter liver disease is often discussed as the hepatic manifestation of the metabolic syndrome [2]. NAFLD is by now the most prevalent liver disease worldwide [3] affecting ~25% of the general global population [4]. In patients with type 2 diabetes, prevalence of NAFLD is even higher, ranging from 30% to 68% [5]. NAFLD is thought to be a rather slowly evolving disease with the development of liver phenotype in most patients ranging from 7 to 14 years per stage of liver fibrosis, and the window of opportunity for treatment seems rather large [6]. However, despite intense research efforts in the last two decades, understanding of mechanisms underlying disease development is still limited, and therapeutic strategies up to now mainly focus on lifestyle interventions.

Overnutrition along with overweight and obesity are acknowledged to be risk factors for the development of NAFLD [7]. Furthermore, results of animal models suggest that macronutrient composition, e.g., a diet rich in saturated fat and/or monosaccharides such as fructose may be critical in the development of NAFLD even in the absence of any marked overnutrition or body weight gain [8,9]. Studies in humans with similar caloric intake also suggest that the dietary intake of patients with NAFLD is markedly different from that of disease-free weight-matched individuals [10]. Indeed, results of these studies suggest that the fiber and polyunsaturated fatty acid (PUFA) intake of NAFLD patients is significantly lower [10]. Furthermore, results of meta-analyses suggest that altering dietary fat intake through supplementing n3 fatty acids, thereby also increasing the ratio of n3 to n6 fatty acids in diet, may improve liver parameters of NAFLD patients [11,12]. Worldwide, soybean oil, having an n3 to n6 ratio of ~1:7.1 (linolenic to linoleic acid) [13,14], is among the most used plant oils in food preparation [14,15]. Results of animal studies assessing the effects of soybean oil on the development of NAFLD are contradictory. While some studies suggest that a diet rich in soybean oil may enhance the development of NAFLD [16,17], others show that compared to the intake of a diet rich in lard, consumption of a diet enriched with soybean oil may lead to lower lipid accumulation in liver [18]. However, the question of whether the addition of small amounts of soybean oil to the diet or fortifying butterfat with small amounts of soybean oil is associated with an increase of dietary intake of n3 fatty acids and an increase of the n3 to n6 ratio that may alter the development of NAFLD has not been studied in depth yet.

Starting from this background, the aim of the present study was to assess whether replacing small amounts of butter fat with soybean oil (4E%) in an otherwise “unhealthy” butterfat-, cholesterol-, and fructose-rich diet affects the development of early signs of non-alcoholic steatohepatitis (NASH), a more progressed stage of NAFLD [19], in mice.

## 2. Materials and Methods

### 2.1. Animals and Treatment

All procedures were approved by the local institutional animal care and use committee (‘Landesamt für Verbraucherschutz’, reference number: 02-004/16, Thuringia, Germany), and animals were handled in accordance with the European Convention for the Protection of Vertebrate Animals Used for Experimental and Other Scientific Purposes. All procedures were carried out with female C57BL/6J mice (8 weeks old) purchased from Janvier (SAS, Le Genest-Saint-Isle, France), as female mice have been shown to be more susceptible to fructose-induced liver damage [20] and to develop early signs of non-alcoholic steatohepatitis (NASH) at a similar rate as male mice [21]. Animals were housed in a pathogen-free barrier facility in groups in standard cages, under controlled conditions accredited by the Association for Assessment and Accreditation of Laboratory Animal Care and had free access to tap water at all times (12 h/12 h light/dark cycle, ~23 °C, ~65% relative humidity). Calculation of number of animals per group was based on previous findings [8,22]. After adaptation to the facility, animals were randomly assigned to three groups (*n* = 6–8/group) and were fed either a liquid control diet (C, 69E% carbohydrates, 12E% fat (soybean oil), 19E% protein; Ssniff, Soest, Germany); a liquid fat-, fructose-, and cholesterol-rich diet (FFC, 60E% carbohydrates, 25E% fat derived from butterfat, 15E% protein with 50% (wt./wt.) fructose and 0.16% (wt./wt.) cholesterol; Ssniff, Soest, Germany); or a liquid fat-, fructose-, and cholesterol-rich diet supplemented with soybean oil (FFC + S, 60E% carbohydrates, 21E% butterfat + 4E% soybean oil with 50% (wt./wt.) fructose and 0.16% (wt./wt.) cholesterol) for 13 weeks, respectively. Based on previous studies of our group, but also others, the length of feeding the different diets was selected as suitable to induce macrovesicular steatosis and early signs of inflammation [8,23], e.g., early signs of NASH, which is a more progressed stage on NAFLD [19]. The composition of diets is summarized in Table S1 and has previously been described in detail [22]. To ensure equal caloric intake, mice fed the fat-, fructose-, and cholesterol-rich diets were pair-fed as previously described [8]. Dietary intake was assessed daily including weekends and public holidays, and body weight was measured weekly. Furthermore, after 12 weeks of feeding, a glucose tolerance test (GTT) was performed under ketamine/xylazine anesthesia after mice had been fasted for 6 h as described in detail before [8]. After 13 weeks of feeding, animals were anesthetized (i.p.) with a mixture of ketamine/xylazine in the morning, blood was collected from the portal vein, and animals were killed by cervical dislocation. Liver and muscle samples from the hindlimb were collected in neutral-buffered formalin or immediately snap-frozen in liquid nitrogen and were stored in a −80 °C freezer for further experiments. Killing and all following measurements were performed in a mixed order of mice.

### 2.2. Cell Culture

J774A.1 cells (DSMZ, Braunschweig, Germany) were cultured at 37 °C in a humidified, 5% carbon dioxide atmosphere with Dulbecco’s modified Eagle medium (DMEM; Pan-Biotech, Aidenbach, Germany) enriched with 1% penicillin/streptomycin (Pan-Biotech, Germany) and 10% fetal bovine serum (FBS, Pan-Biotech, Aidenbach, Germany). Then, 10 mM fatty acid (dissolved in 0.1 M NaOH)/1% bovine serum albumin (BSA) stock solutions were prepared as previously described [24,25]. At 70–80% confluence, cells were pre-incubated for 2 h with DMEM (10% FBS and 1% penicillin/streptomycin) containing 1.2 mM NaOH (vehicle) or 0.6 and 1.2 mM fatty acids (mix of linolenic acid:linoleic acid (1:7.1), respectively, both Sigma-Aldrich Chemie GmbH, Steinheim, Germany), followed by stimulation with 0 or 50 ng/mL lipopolysaccharide (LPS) for 18 h. Cells were then harvested with peqGOLD TriFast (VWR, Darmstadt, Germany) and stored at −80 °C for subsequent RNA isolation.

### 2.3. Evaluation of Liver Damage and Inflammation

Paraffin-embedded liver sections (4 µm) were stained with hematoxylin and eosin (Sigma Aldrich Chemie GmbH, Steinheim, Germany) to evaluate liver histology using the NAFLD activity score (NAS) as detailed by others [26]. Alanine transaminase (ALT) and aspartate transaminase (AST) activity in the plasma of mice was determined in a routine laboratory (University Hospital of Jena, Jena, Germany). Neutrophil granulocytes were stained in liver sections using a commercially available kit (Naphthol AS-D Chloroacetate kit, Sigma-Aldrich Chemie GmbH, Steinheim, Germany) as detailed previously [27]. Immunohistochemical staining was used to assess 4-hydroxynonenal (4-HNE) protein adducts and F4/80-positive cells in liver sections as described previously [27,28]. To determine the extent of 4-HNE staining in liver sections, an image acquisition and analysis system incorporated in the microscope (Leica DM4000 B LED, Leica, Wetzlar, Germany) was used. Staining detected in eight microscopic fields (200× magnification) of each liver section was assessed to determine means. The number of neutrophil granulocytes and F4/80-positive cells were counted in eight microscopic fields in each tissue section (200× magnification) using a camera integrated in a microscope (Leica DM4000 B LED, Leica, Wetzlar, Germany) as described previously [27,28].

### 2.4. Endotoxin and Free Fatty Acid (FFA) Measurements, Tumor Necrosis Factor α (TNF-α) ELISA, and Peroxisome Proliferator-Activated Receptor γ (PPARγ) Transcription Factor Assay

Bacterial endotoxin levels in portal plasma were measured using a limulus amebocyte lysate assay as detailed previously [29]. The concentration of FFA in plasma was measured with a commercially available kit (Free Fatty Acid Quantitation Kit, Sigma-Aldrich Chemie GmbH, Steinheim, Germany). Protein concentration of TNF-α in liver homogenate was determined using a commercially available TNF-α ELISA (Assaypro, St. Charles, MO, USA). Whole-cell extract of liver obtained with a commercially available nuclear extract kit (Active motif, La Hulpe, Belgium) was used to measure the activity of PPARγ (PPAR-γ Transcription Factor Assay Kit, Active Motif, La Hulpe, Belgium).

### 2.5. Western Blot Analysis

To determine protein levels of occludin and β-actin in proximal small intestine, protein lysates were obtained using Trizol (peqGOLD Trifast; Darmstadt, Germany) as previously described [30]. Protein lysates (1 µg/µL for occludin and 0.1 µg/µL for β-actin) were separated in a sodium dodecyl sulfate–polyacrylamide gel and transferred to a Hybond^TM^-P polyvinylidene difluoride membrane (Bio-Rad Laboratories, USA). Membranes were further incubated with specific primary antibodies (occludin (Thermo Fisher Scientific, Waltham, MA, USA) and β-actin (Cell Signaling Technology, Massachusetts, USA)) and their respective secondary antibodies. Detection of occludin and β-actin had to be performed on two separated membranes to avoid overstaining of β-actin. For the detection of protein bands, the Super Signal Western Dura kit (Thermo Fisher Scientific, Waltham, MA, USA) was used, and densitometric analyses were performed using ChemiDoc XRS System (Bio-Rad Laboratories, Hercules, CA, USA) as detailed previously [31].

### 2.6. Assessment of Arginase and Myeloperoxidase (MPO) Activity as Well as Griess Assay

Arginase activity in liver tissue was assessed using the modified method described by Corraliza et al. [32] and detailed previously [33]. MPO activity was determined in homogenized liver tissue as previously detailed [34] adapted to mice hepatic tissue (~50 mg tissue homogenized in 300 μL Dulbecco’s Phosphate Buffered Saline (DPBS, PAN Biotech, Aidenbach, Germany)), and normalized to protein concentration. Nitric oxide (NOx) concentration was measured in the cell supernatant using the Griess reagent assay (Promega, Mannheim, Germany).

### 2.7. RNA Isolation and Real-Time Polymerase Chain Reaction (RT-PCR)

Using peqGOLD Trifast, RNA from liver and muscle tissue as well as cells was extracted. After measuring concentration of RNA, cDNA was synthetized with a reverse-transcription system. RT-PCR was performed using primers listed in Table S2, and the expression of the respective genes normalized to 18S was evaluated as previously described [22].

### 2.8. Statistical Analysis

All data are presented as means ± standard error of mean (SEM). Using PRISM (version 7.03, GraphPad Software, San Diego, CA, USA), a Grubb’s outlier test was performed for all measurements before further statistical analysis. For all data, a one-factorial analysis of variance (ANOVA) followed by Tukey’s post hoc test was carried out to determine significant differences between all groups (*p* ≤ 0.05). Bartlett’s test was used to assess homogeneity of variance, and in case of inhomogeneity of variances, data were log-transformed.

## 3. Results

### 3.1. Markers of Liver Damage and Inflammation in Mice Fed a Butterfat-Rich FFC ± Soybean Oil

**Figure 1:**
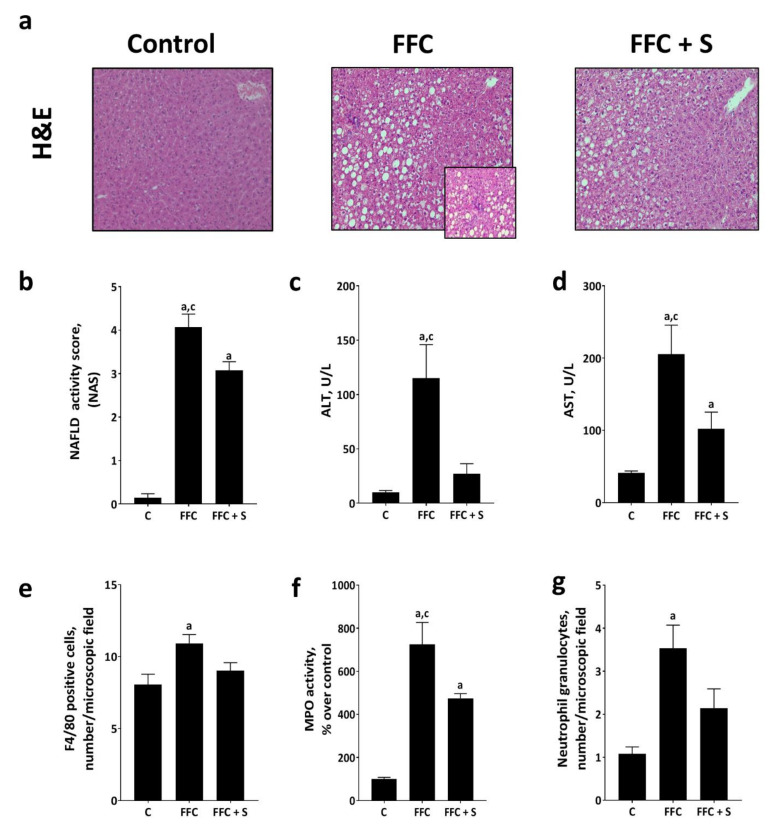
Effect of fortifying the fat-, fructose-, and cholesterol-rich diet with 4E% soybean oil on markers of liver damage in mice. (**a**) Representative pictures (100×, 400×) and (**b**) evaluation of hematoxylin and eosin (H&E) staining using NAFLD activity score (NAS) of liver sections. (**c**) Alanine transaminase (ALT) and (**d**) aspartate transaminase (AST) activity in plasma, (**e**) number of F4/80-positive cells per microscopic field in liver tissue, (**f**) myeloperoxidase (MPO) activity in liver tissue, and (**g**) number of neutrophil granulocytes per microscopic field in liver tissue. Data are presented as means ± SEM, *n* = 6–8 mice per group with *n* = 6–8 livers being analyzed per group and measurements. Data of NAS, ALT, AST, MPO, and neutrophil granulocytes were log-transformed before statistical analysis. C-group ALT only *n* = 4 per group as values were in part below the level of detection. C: control diet; FFC: fat-, fructose-, and cholesterol-rich diet; FFC + S: fat-, fructose-, and cholesterol-rich diet supplemented with soybean oil. ^a^ *p* ≤ 0.05 compared with mice fed the C-diet. ^c^ *p* ≤ 0.05 compared with mice fed the FFC + S–diet.

Despite similar caloric intake and body weight gain (Table 1), fat accumulation and signs of inflammation in liver tissue as assessed by NAS were significantly less pronounced in FFC + S–fed mice than in those being fed the “normal” FFC (Figure 1 and Table 1). Ballooning cells were not very prevalent in either of the FFC-fed groups regardless of the fat composition of diets (Figure 1 and Table 1). In line with these findings, activities of ALT and AST in plasma as well as the liver to body weight ratio were also significantly lower in FFC + S–fed mice when compared to FFC-fed mice (*p* ≤ 0.05 for all comparisons) (Figure 1 and Table 1). Nevertheless, despite being lower than in FFC-fed mice, NAS, AST activity in plasma and liver to body weight ratios of FFC + S–fed mice were significantly higher than in C-fed animals (*p* ≤ 0.05), while ALT activity in plasma was at the level of controls. The numbers of F4/80-positive cells and neutrophiles were also significantly higher in the livers of FFC-fed mice than in those of C-fed mice (*p* ≤ 0.05) (Figure 1 and Figure S1), whereas numbers of these cells in livers of FFC + S–fed mice differed neither from those of FFC- nor of C-fed mice. MPO activity was significantly higher in the livers of FFC-fed mice than in those of the C-fed and FFC + S–fed animals (*p* ≤ 0.05). Still, MPO activity in the liver tissue of FFC + S–fed animals was significantly higher than in livers of C-fed mice (*p* ≤ 0.05) (Figure 1). The hepatic protein concentration of TNF-α did not differ between groups (Table 1). Arginase activity in livers was similar between FFC- and FFC + S–fed mice but significantly lower than in livers of C-fed mice (*p* ≤ 0.05) (Table 1).

### 3.2. Glucose Tolerance and Markers of Glucose Metabolism in Liver and Muscle in Mice Fed a Butterfat-Rich FFC ± Soybean Oil

**Figure 2:**
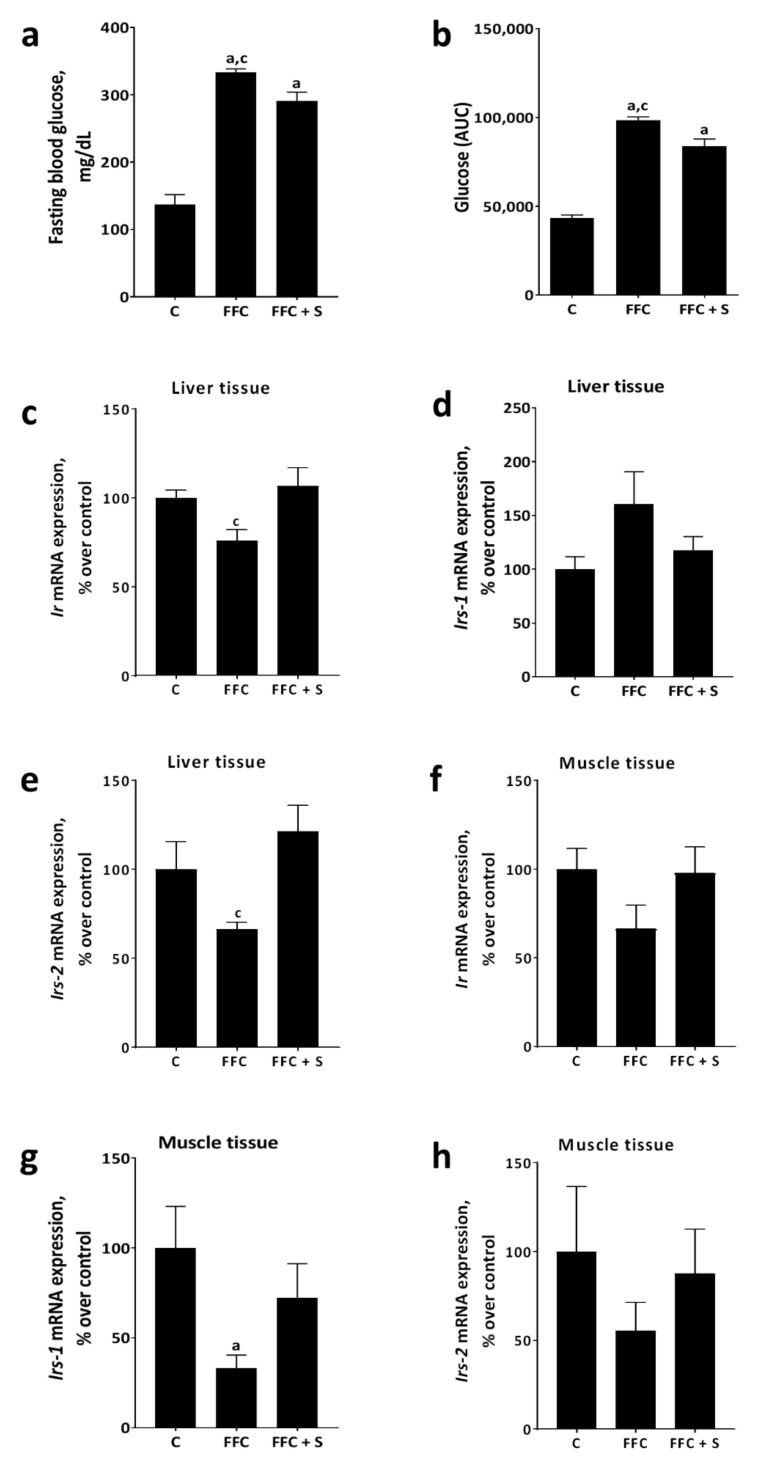
Effect of fortifying the fat-, fructose-, and cholesterol-rich diet with 4E% soybean oil on markers of glucose metabolism in mice. (**a**) Fasting blood glucose levels and (**b**) area under the curve (AUC) of glucose levels during the glucose tolerance test. Expression of (**c**) *insulin receptor* (*Ir*), (**d**) *insulin receptor substrate-1* (*Irs-1*), and (**e**) *insulin receptor substrate-2* (*Irs-2*) mRNA in the liver tissue. Expression of (**f**) *Ir*, (**g**) *Irs-1*, and (**h**) *Irs-2* mRNA in muscle tissue. Data are presented as means ± SEM, *n* = 6–8 mice per group with *n* = 6–8 livers or muscle specimens being analyzed per group and measurements. Data of AUC, *Irs-1* and *Irs-2* in liver tissue, and *Irs-1* in muscle tissue were log-transformed before statistical analysis. C: control diet; FFC: fat-, fructose-, and cholesterol-rich diet; FFC + S: fat-, fructose-, and cholesterol-rich diet supplemented with soybean oil. ^a^ *p* ≤ 0.05 compared with mice fed the C-diet. ^c^ *p* ≤ 0.05 compared with mice fed the FFC + S–diet.

Fasting blood glucose and area under the curve (AUC) of GTT were significantly higher in FFC-fed mice when compared to FFC + S– and C-fed animals (*p* ≤ 0.05); however, despite being lower than in FFC-fed mice, both fasting glucose levels and AUC of GTT were significantly higher in FFC + S–fed mice than in C-fed animals (*p* ≤ 0.05) (Figure 2 and Figure S2). Furthermore, the mRNA expression of *insulin receptor (Ir)* and *insulin receptor substrate 2 (Irs-2)* were significantly lower in livers of FFC-fed mice when compared to that in the FFC + S–fed animals (*p* ≤ 0.05) (Figure 2), with the latter being almost at the level of C-fed mice. Hepatic mRNA expression of *insulin receptor substrate 1 (Irs-1)* was similar between groups (Figure 2). In muscle tissue, mRNA expression of *Ir* and *Irs2* was similar between groups. In contrast, mRNA expression of *Irs*-*1* was significantly lower in FFC-fed mice compared to that in C-fed animals (*p* ≤ 0.05). Differences alike were not found when comparing FFC + S– and C-fed mice (Figure 2).

### 3.3. Markers of Lipogenesis in Liver Tissue of Mice Fed a Butterfat-Rich FFC ± Soybean Oil

Expressions of *sterol regulatory element-binding protein 1c* (*Srebp-1c*), *acetyl-CoA carboxylase* (*Acc*), and *stearoyl-CoA desaturase-1* (*Scd-1*) as well as *carnitine palmitoyltransferase 1* (*Cpt1*) mRNA in liver tissue were similar between FFC- and FFC + S–fed mice, and in the case of *Acc* and *Scd-1* mRNA expression in livers of the FFC-fed groups were significantly higher than in livers of C-fed animals (*p* ≤ 0.05) (Table 2), while in the case of *Cpt1* only the FFC-fed group was significantly higher than C-fed mice, as data varied considerably within group. Expression of *acyl-CoA oxidase 1* (*Acox1*) mRNA was significantly higher in the livers of FFC-fed animals compared to that in FFC + S–fed animals (*p* ≤ 0.05) and by trend when comparing FFC-fed mice to C-fed animals (*p* = 0.06) (Table 2). No differences regarding *Acox1* expression were found between FFC + S– and C-fed mice (Table 2). *Fatty acid synthase* (*Fas*) mRNA expression was significantly higher in both FFC-fed groups compared to that in C-fed animals (*p* ≤ 0.05) and by trend when comparing FFC- and FFC + S–fed mice (*p* = 0.07) (Table 2). Furthermore, PPARγ activity was significantly higher in the livers of FFC-fed mice compared to C-fed mice (*p* ≤ 0.05). Similar differences were not observed when comparing FFC + S– and C-fed mice as data varied considerably (Table 2). Furthermore, expression of *G protein-coupled receptor 120* (*Gpr120*) was significantly higher in the livers of FFC-fed mice compared to that in C- and FFC + S–fed mice (*p* ≤ 0.05) (Table 2). Moreover, while being lower than in FFC-fed mice, *Gpr120* mRNA expression in the livers of FFC + S–fed mice was still significantly higher than in C-fed animals (*p* ≤ 0.05) (Table 2). Interestingly, concentrations of FFA in plasma were similar between groups (Table 2).

### 3.4. Bacterial Endotoxin Levels in Portal Vein and Markers of Lipid Peroxidation and Endotoxin-Dependent Signaling Cascades in Livers of Mice Fed a Butterfat-Rich FFC ± Soybean Oil

**Figure 3:**
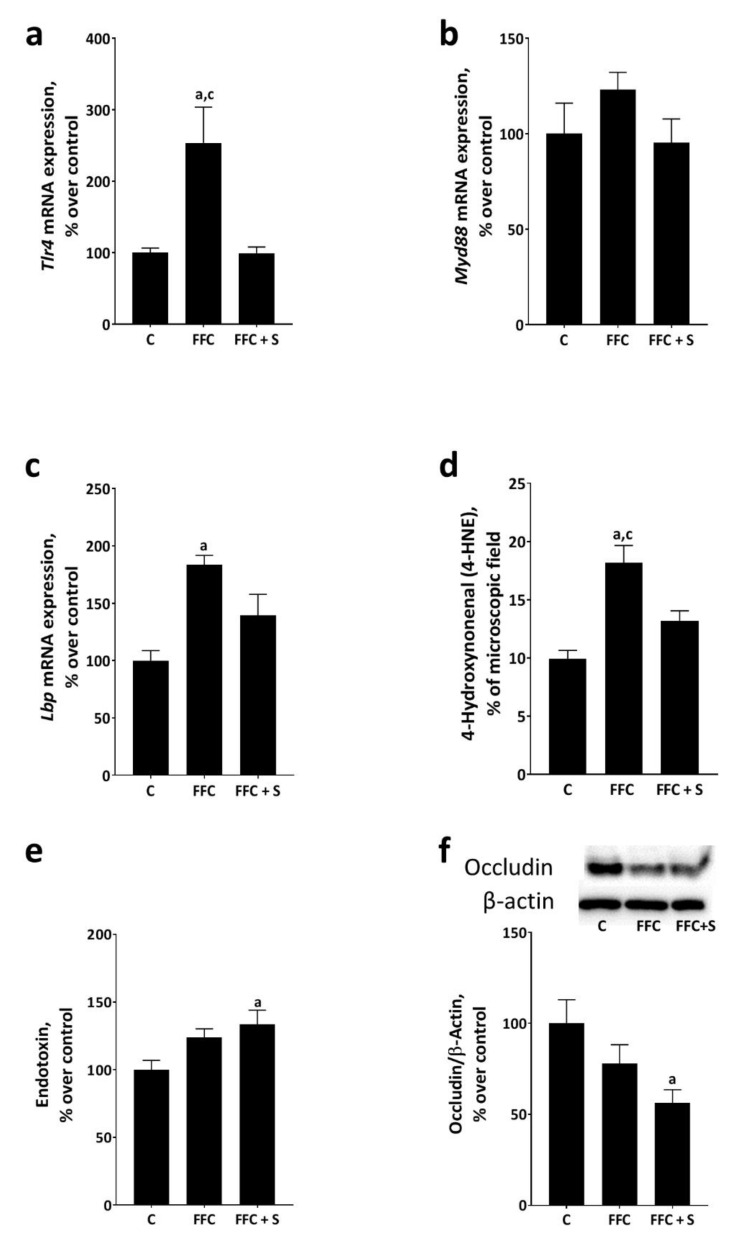
Effect of fortifying the fat-, fructose-, and cholesterol-rich diet with 4E% soybean oil on portal endotoxin levels and markers of TLR4 signaling in the liver tissue of mice. Hepatic mRNA expression of (**a**) *toll-like receptor 4* (*Tlr4*), (**b**) *myeloid differentiation primary response 88 (Myd88)*, (**c**) *lipopolysaccharide binding protein (Lbp)*, (**d**) densitometric analysis of 4-hydroxynonenal (4-HNE) protein adduct staining in liver sections, (**e**) portal plasma endotoxin concentration, and (**f**) protein levels of occludin normalized to beta-actin (β-actin). Data are presented as means ± SEM, *n* = 6–8 mice per group with *n* = 6–8 livers being analyzed per group and measurements. Data of *Tlr4* were log-transformed before statistical analysis. C: control diet; FFC: fat-, fructose-, and cholesterol-rich diet; FFC + S: fat-, fructose-, and cholesterol-rich diet supplemented with soybean oil. ^a^ *p* ≤ 0.05 compared to C. ^c^ *p* ≤ 0.05 compared to FFC + S.

Expression of *toll-like receptor 4 (Tlr4)* was significantly higher in the livers of FFC-fed mice than in livers of C- and FFC + S–fed mice (*p* ≤ 0.05) (Figure 3). *Tlr4* mRNA expression was similar in livers of C- and FFC + S–fed mice. Expression of *myeloid differentiation primary response 88* (*Myd88)* mRNA was similar between groups regardless of further treatments as expression varied considerably (Figure 3). Expression of *lipopolysaccharide binding protein (Lbp)* was significantly higher in the livers of FFC-fed mice compared to that in C-fed mice (*p* ≤ 0.05) and by trend compared to FFC + S–fed mice (*p* = 0.08) (Figure 3). Furthermore, concentrations of 4-HNE protein adducts, shown to be also triggered in liver tissue through TLR4-dependent signaling pathways in settings of NAFLD [35], were significantly higher in the liver tissue of FFC-fed mice than in C- and FFC + S–fed mice (*p* ≤ 0.05) (Figure 3 and Figure S1). Again, differences alike were not found when comparing concentrations of 4-HNE protein adducts in liver tissue between C- and FFC + S–fed mice. Somewhat contrasting these findings, levels of endotoxins in portal plasma were significantly higher in FFC + S–fed mice than in controls (*p* ≤ 0.05) (Figure 3). Due to interindividual variations, similar differences were not found when comparing portal endotoxin levels of FFC- and C-fed mice (Figure 3). In line with these findings, occludin protein levels in the proximal small intestine were also significantly lower in FFC + S–fed mice than in C-fed animals (*p* ≤ 0.05) (Figure 3). Again, due to the high variability within groups, the level of significance was not reached when comparing occludin protein in small intestinal tissue between FFC- and C-fed mice (Figure 3).

### 3.5. Effect of Linolenic and Linoleic Acid, in a Ratio Equivalent to That Found in Soybean Oil, on LPS-Dependent Activation of J774A.1 Cells

**Figure 4:**
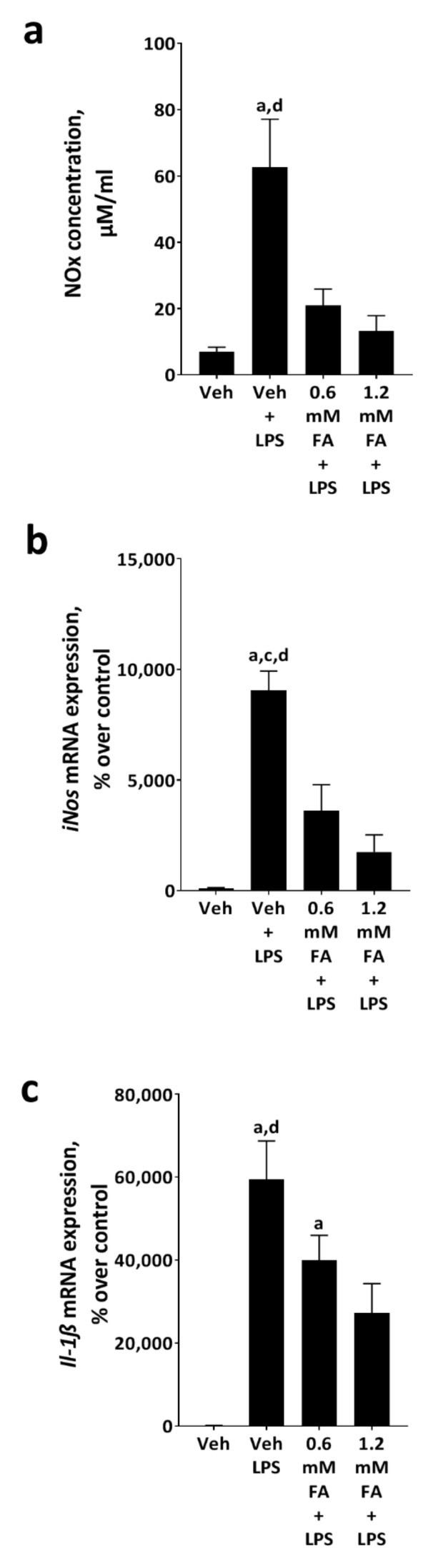
Effect of a 0.6 and 1.2 mM linolenic and linoleic acid mix, respectively, on the LPS-induced activation of J774A.1 cells. (**a**) NOx concentration in cell culture medium and mRNA expression of (**b**) *inducible nitric oxide synthase* (*iNos*) as well as (**c**) *interleukin 1 β* (*Il-1β*) in cells. Cells were pre-incubated with a linolenic acid:linoleic acid mix (1:7.1) for 2 h followed by an 18 h incubation with 0 or 50 ng/mL LPS. Data are presented as means ± SEM, *n* = 3–5. Veh: vehicle; Veh + LPS: vehicle challenged with lipopolysaccharide; FA + LPS: fatty acids plus lipopolysaccharide. ^a^ *p* ≤ 0.05 compared to Veh. ^c^ *p* ≤ 0.05 compared to 0.6 mM FA + LPS. ^d^ *p* ≤ 0.05 compared to 1.2 mM FA + LPS. Data of NOx concentration were log-transformed before statistical analysis.

To further determine whether the effects found on the TLR4 signaling cascade in vivo were related to linolenic and linoleic acids found in soybean oil, murine monocytes (J774A.1 cells), used as a model of Kupffer cells, were pre-incubated with these fatty acids in a ratio found in soybean oil (1:7.1; linolenic acid:linoleic acid) and further stimulated with LPS. As exposure to fatty acids alone had no effects on any of the parameters assessed in the present study, results of cells only exposed to vehicle are shown as representatives of all control groups. Concentration of NOx in media and mRNA expressions of *inducible nitric oxide synthase* (*iNos*) and *interleukin-1 beta* (*Il-1β*) in cells only exposed to LPS were significantly higher than in vehicle-treated cells (*p* < 0.05) (Figure 4). The pre-incubation of cells with 0.6 mM and even more so with 1.2 mM of the fatty acid mix significantly attenuated the LPS-dependent increase in *iNOS* mRNA expression (*p* < 0.05) (Figure 4). NOx in media and expression of *Il-1β* were only significantly attenuated in cells pre-incubated with 1.2 mM of the fatty acid mix compared to cells only exposed to LPS (*p* < 0.05) (Figure 4). However, *Il-1β* mRNA expression of cells treated with 0.6 mM of the fatty acid mix was still significantly higher than that of vehicle-treated cells (*p* < 0.05) (Figure 4).

## 4. Discussion

Insulin resistance and NAFLD are among the most common non-communicable disease worldwide [3,4,5,36]. Despite intense research efforts throughout the last decades, universally accepted drug-based therapies for the treatment of NAFLD are still lacking and lifestyle interventions, e.g., focusing on a weight reduction and increase of physical activity are still first line treatments. Herein, a reduction of general fat intake along with an increase of the intake of plant-derived oils is often among the key recommendations [37,38]. However, to date, knowledge on the effects of fortifying the diet of NAFLD patients with selected plant oils is limited and recommendations often focused on olive oil as part of the Mediterranean diet [38,39]. In the present study, employing a pair-feeding liquid diet model allowing for an iso-caloric feeding of animals, we showed that fortifying a butterfat-, fructose-, and cholesterol-rich diet with soybean oil (4E%) in part attenuated the development of early NASH in mice. Indeed, macrovesicular fat accumulation and inflammation in liver tissue as well as high transaminase activity in the blood, found to be elevated in mice fed the “normal” FFC, were markedly attenuated in animals receiving the FFC fortified with soybean oil. Nevertheless, the addition of the plant oil to the diet did not attenuate the development of NAFLD completely but rather seemed to “slow” it down, as TNF-α protein concentration was not altered and several markers were still higher than in C-fed mice. The protective effects of fortifying the diet with soybean oil were also associated with less severe signs of glucose intolerance, e.g., lower fasting glucose levels and AUC of GTT. Similar to indexes of liver damage, most of these markers were still not at the level of controls. Markers of insulin signaling such as *Ir* and *Irs-2*, shown to be altered in livers of humans and rodents of NAFLD [40,41], were also significantly lower in the livers of FFC-fed mice when compared to FFC + S–fed animals. *Irs-1* mRNA expression in muscle tissue of FFC-fed mice was significantly lower than in C-fed group while similar differences were not found compared to FFC + S–fed animals. Similar differences between groups were not found, when assessing expression of *Irs-1* in liver tissue and *Ir* and *Irs-2* mRNA expression in muscle tissue. The lack of marked differences between groups in the expression of these genes in muscle might have resulted from the marked variability in expression of these genes within groups. However, our results in part contrast those of others reporting that in ob/ob mice suffering from both NAFLD and impaired glucose tolerance protein expressions of both *Irs-1* and *-2* in the liver and muscle tissue were lower than in littermates [41,42]. Differences between the findings of others and our findings might have resulted from the lack of abnormal body weight gain in the present study. Indeed, alterations in the expression of *Ir*, *Irs-1*, and *Irs-2* have been shown before to be related to the presence of overweight [43]. Results of our study also in part contrast those of others assessing the effects of soybean oil on the development of NAFLD [16]. For instance, in the study of Henkel et al., feeding mice a 49E% soybean-oil-enriched high-fat diet was associated with a more pronounced hepatic injury and insulin resistance as well as higher expression of pro-inflammatory cytokines in the liver, when compared to a lard-enriched high-fat diet [16]. However, Zhao et al. recently reported that the addition of 40E% soybean oil to a high-fat diet in rats had beneficial effects regarding hepatic fat accumulation, inflammation, and insulin resistance [18], further suggesting, that the amount of soybean oil added to the diet might be critical. Indeed, results of others also suggest that, when being fed in high amounts, PUFAs may exert adverse health effects (for overview see [44,45]). For instance, increased intake of PUFAs has been linked to high plasma indices of oxidative stress and of lipid peroxidation [46,47]. Taken together, results of the present study suggest that fortifying a butterfat-, fructose-, and cholesterol-rich diet with soybean oil attenuates the development of glucose intolerance and early NASH, e.g., the development of macrovesicular steatosis and inflammation. However, whether fortifying the diet with soybean oil also has beneficial effects on the development of more severe stages of the liver disease, e.g., fibrosis and the development of type 2 diabetes as well as whether enriching the diet with soybean oil also has beneficial effects in humans and the effective doses needed remain to be determined.

### 4.1. The Protective Effects of Enriching the Diet with Soybean Oil Are Not Associated with Changes of Markers of Lipogenesis

Despite observations of others suggesting that soybean oil may impact markers of lipogenesis in liver differently from fats obtained from animal sources [18,48], in the present study, mRNA expressions of *Srebp-1c* was not altered while mRNA expression of *Acc*, *Scd-1*, and *Fas* was altered in livers of both, FFC- and FFC + S–fed mice, to a similar extent when compared to C-fed mice. As data varied considerably, *Cpt1* mRNA expression was only significantly higher in FFC-fed mice compared to C-fed animals, while not reaching the level of significance when comparing FFC + S– and C-fed mice. Differences between the findings of other groups [18,48] and our own study might have resulted from differences in model organisms used, e.g., mice in the present study vs. rats and fish, respectively, in the study of others as well as the amount of soybean oil used (present study: 4E% vs. 29E% and 20%, respectively, in the study of others) [48,49]. Despite the lack of difference in *Srebp-1c*, mRNA expressions of *Fas* and *Acox1* were on trend or significantly higher in the livers of FFC-fed mice than in FFC + S–fed animals. Indeed, it has been shown that *Srebp-1c*, being a key regulator of genes involved in lipogenesis [50], is not the only regulator of *Fas* and *Acox1* mRNA expression [50,51,52]. Additionally, mRNA expression of *Gpr120*, shown to mediate the anti-inflammatory effects of docosahexaenoic acid (DHA) in mice with methionine–choline-deficient diet-induced NAFLD [53], was significantly higher in the livers of both FFC-fed groups compared to controls. Somewhat surprisingly and contrasting the findings for inflammation suggesting that *Gpr120* mediated the anti-inflammatory effects of PUFA [54], *Gpr120* mRNA expression was significantly more pronounced in FFC-fed mice than in FFC + S–fed animals. Furthermore, the activity of PPARγ in liver tissue, shown to be downstream of *Gpr120* and to be involved in mediating its anti-inflammatory effects [55], was only significantly higher in the FFC-fed group compared to C-fed mice in the present study. Reasons for this apparent lack of responsiveness of both *Gpr120* mRNA expression and PPARγ activity need to be determined in future studies. However, it could be that the amount of soybean oil added to the diet (4E%) in the present study was not sufficient to induce the changes observed by others before [48,49]. Indeed, Nakamoto et al. used plain DHA (50 μL/day) [53], whereas in the present study, 4E% soybean oil was added to the FFC diet. Somewhat surprisingly, levels of plasma FFA were similar between groups, contrasting the findings of others in settings of diet-induced NAFLD in rodents [56]. However, in line with our findings, results of others also suggest that in the absence of abnormal weight gain and overweight, levels of FFA in plasma of mice fed a high-fat and high-sugar diet remain unchanged [57]. Taken together, results in the present study suggest that the beneficial effects of supplementing soybean oil were not primarily associated with its effect on lipogenesis but rather with other mechanisms. Nevertheless, results of the present study by no means preclude that different concentrations or composition of PUFAs may alter markers of de novo lipogenesis in the liver.

### 4.2. How Does the Addition of Soybean Oil Attenuate the Development of Diet-Induced NAFLD in Mice?

Results of our own and other groups suggest that intestinal barrier dysfunction and subsequently an increased translocation of bacterial endotoxin and induction of TLR4-dependent signaling cascades may be critical in the onset and progression of both NAFLD and insulin resistance [35,58,59]. In the present study, despite higher plasma endotoxin levels in the portal vein and lower occludin protein levels in the small intestine, mRNA expression of *Lbp* and *Tlr4* in the livers of FFC + S–fed mice were by trend and significantly lower when compared to FFC-fed animals, reaching almost the level of controls. The lack of differences for endotoxin portal plasma levels and occludin protein levels when comparing C- and FFC-fed mice was related to interindividual differences within groups. *Myd88* mRNA expression was similar between groups, as data varied considerably within groups, differences did not reach the level of significance. Moreover, it has been shown before by others that Myd88 is regulated not only at the level of mRNA but also through phosphorylation [60]. In support of these findings, levels of 4-HNE protein adducts, being a marker of lipid peroxidation [61] and shown before to also be triggered through endotoxin- and TLR4-dependent pathways in liver tissue [62], were almost at the level of C-fed animals in livers of FFC + S–fed animals. These data suggest that intestinal barrier dysfunction, shown before to be associated with the development of NAFLD (for overview see [63]), was not affected by the addition of soybean oil to the diet. However, together with the data obtained in cell culture experiments in the present study, our data suggest that the addition of soybean oil to the diet may attenuate TLR4 signaling in the liver/immune cells. Indeed, in the present study, the addition of linolenic and linoleic acid in a ratio of 1:7.1 to the cell culture media attenuated the LPS-dependent induction of *iNos* and *Il-1*β mRNA expression as well as the increase in NOx levels in an almost dose-dependent manner. These findings are in line with those from other studies reporting that the addition of PUFAs such as n-3 polyunsaturated fatty acids to cell culture media attenuated the M1-like activation of macrophage-like cells and of NF-kappa B-dependent signaling pathways [64], e.g., the increase of pro-inflammatory cytokines such as IL-1β and the formation of reactive oxygen and even more so reactive nitrogen species [65,66].

Taken together, fortifying the diet or cell culture media with small amounts of PUFAs seems to attenuate the LPS-dependent induction of the TLR4 signaling cascade in liver and immune cells. While results of the present study suggest that this is related to an attenuation of the LPS-dependent induction of iNOS, further studies are needed to determine the molecular mechanisms involved.

## 5. Conclusions

Taken together, results of the present study suggest that an addition of small amounts of soybean oil (e.g., 4E%) to a butterfat-, fructose-, and cholesterol-rich diet is sufficient to at least partially attenuate the development of diet-induced insulin resistance and NAFLD in mice. Our results further suggest that the beneficial effects of fortifying the diet with soybean oil may be related to an attenuation of the LPS-dependent induction of the TLR4 signaling cascade in liver tissue. However, further studies are needed to determine long-term effects and optimal doses. In addition, whether fortifying dietary fat (e.g., butterfat) with plant-derived oils such as soybean oil also attenuates the development of NAFLD and insulin resistance in humans or even more so possesses therapeutic effects remains to be determined.

## Figures and Tables

**Table 1:** Effect of fortifying a fat-, fructose-, and cholesterol-rich diet with 4E% soybean oil on food intake, body weight, and parameters of liver damage in mice.

Parameter	Diet Groups		
	C	FFC	FFC + S
Caloric intake, kcal/g body weight/day	0.39 ± 0.01	0.44 ± 0.01 ^a^	0.43 ± 0.01 ^a^
Body weight, g	21.6 ± 0.5	23.9 ± 0.6 ^a^	23.8 ± 0.7 ^a^
Weight gain, g	3.6 ± 0.3	5.8 ± 0.4 ^a^	5.5 ± 0.5 ^a^
Liver weight, g *	0.9 ± 0.03	1.7 ± 0.13 ^a,c^	1.3 ± 0.05 ^a^
Liver to body weight ratio, % *	4.1 ± 0.09	7.1 ± 0.36 ^a,c^	5.7 ± 0.08 ^a^
Steatosis, NAS	0.3 ± 0.1	2.4 ± 0.2 ^a,c^	1.9 ± 0.1 ^a^
Inflammation, NAS	0 ± 0	1.6 ± 0.1 ^a,c^	1.0 ± 0.1 ^a^
Ballooning	0 ± 0	0.04 ± 0.02	0.04 ± 0.02
TNF-α, pg/mg protein *	28.8 ± 3.7	39.5 ± 4.0	33.6 ± 0.9
Arginase activity, % over control *	100 ± 14	37.4 ± 2.3 ^a^	50.3 ± 7.1 ^a^

Values are means ± SEM, *n* = 6–8 mice per group with *n* = 6–8 livers being analyzed per group and measurements. ^a^ *p* ≤ 0.05 compared to C. ^c^ *p* ≤ 0.05 compared to FFC + S. * Data of liver weight, liver to body weight ratio, TNF-α and arginase activity were log-transformed before statistical analysis. C, control diet; FFC, fat-, fructose-, and cholesterol-rich diet; FFC + S, fat-, fructose- and cholesterol-rich diet supplemented with soybean oil, NAFLD, non-alcoholic fatty liver disease; NAS, NAFLD activity score; TNF-α, tumor necrosis factor α.

**Table 2:** Effect of fortifying the fat-, fructose-, and cholesterol-rich diet with 4E% soybean oil on hepatic markers of lipid metabolism and inflammation in mice.

Parameter	Diet Groups		
	C	FFC	FFC + S
*Srebp-1c* mRNA expression, % of control	100 ± 9.5	130 ± 17	117 ± 12
*Acc* mRNA expression, % of control *	100 ± 18	446 ± 75 ^a^	428 ± 78 ^a^
*Scd-1* mRNA expression, % of control *	100 ± 9.8	393 ± 67 ^a^	355 ± 80 ^a^
*Acox1* mRNA expression, % of control	100 ± 12	135 ± 7.8 ^c^	91.5 ± 9.0
*Cpt1* mRNA expression, % of control	100 ± 14	148 ± 9.1 ^a^	130 ± 11
*Fas* mRNA expression, % of control *	100 ± 15	617 ± 65 ^a^	386 ± 63 ^a^
PPARγ activity, % of control *	100 ± 2.0	150 ± 19 ^a^	123 ± 9.3
*Gpr120* mRNA expression, % of control	100 ± 14	242 ± 11 ^a,c^	180 ± 23 ^a^
FFA, nmol/µL	0.47 ± 0.06	0.33 ± 0.03	0.42 ± 0.03

Values are means ± SEM, *n* = 6–8 mice per group with *n* = 6–8 livers being analyzed per group and measurements. ^a^ *p* ≤ 0.05 compared to C. ^c^ *p* ≤ 0.05 compared to FFC + S. * Data of *Acc*, *Scd-1*, *Fas*, and PPARγ activity were log-transformed before statistical analysis. Acc, acetyl-CoA carboxylase; Acox1, acyl-CoA oxidase 1; C diet, control diet; Cpt1, carnitine palmitoyltransferase 1; Fas, fatty acid synthase; FFA, free fatty acids; FFC, fat-, fructose-, and cholesterol-rich diet; FFC + S, fat-, fructose-, and cholesterol-rich diet supplemented with soybean oil; *Gpr120*, G protein-coupled receptor 120; Scd-1, stearoyl-CoA desaturase-1; Srebp-1c, sterol regulatory element-binding protein 1c; PPARγ, peroxisome proliferator-activated receptor **γ**.

## Supplementary Materials

The following are available online at https://www.mdpi.com/2072-6643/13/3/959/s1, Table S1: Composition of diets (Ssniff Spezialdiäten GmbH, Soest, Germany). Table S2: Primer sequences. Figure S1: Representative pictures of (a) F4/80-positive cells (200×), (b) 4-hydroxynonenal (4-HNE) protein adduct staining (200×) and neutrophil granulocyte (200×) in liver sections of C-, FFC-, and FFC + S–fed mice. C: control diet; FFC: fat-, fructose-, and cholesterol-rich diet; FFC + S: fat-, fructose-, and cholesterol-rich diet supplemented with soybean oil. ^a^ *p* ≤ 0.05 compared to C. ^c^ *p* ≤ 0.05 compared to FFC + S. Figure S2: Glucose tolerance test of C-, FFC-, and FFC + S–fed mice. C: control diet; FFC: fat-, fructose-, and cholesterol-rich diet; FFC + S: fat-, fructose-, and cholesterol-rich diet supplemented with soybean oil. ^a^ *p* ≤ 0.05 compared to C. ^c^ *p* ≤ 0.05 compared to FFC + S.
