# Supplementary material for: Fortifying Butterfat with Soybean Oil Attenuates the Onset of Diet-Induced Non-Alcoholic Steatohepatitis and Glucose Intolerance
Source: Nutrients. 2021 Mar 16;13(3):959. doi: 10.3390/nu13030959 (PMC8001628; doi:10.3390/nu13030959)
Supplement: Supplementary file 1 [file nutrients-13-00959-s001.pdf]

Table S1: Composition of diets (Ssniff Spezialdiäten GmbH, Soest, Germany).

		Control	FFC	FFC+S
**Metabolizable energy**	MJ/kg	15.7	17.8	17.8
**Carbohydrate**	kcal %	69	60	60
**Fat**	kcal %	12	25	25
**Protein**	kcal %	19	15	15
**Soybean oil**	kcal %	12		4
**Butterfat**	Kcal %		25	21
**Fructose**	% wt/wt		50	50
**Cholesterol**	% wt/wt		0.16	0.16

FFC, fat-, fructose-, and cholesterol-rich diet; FFC+S, fat-, fructose-, and cholesterol-rich diet supplemented with soybean oil.

Table S2: Primer sequences

	Forward (5′- 3′)	Reverse (5′-3′)	Accession Number
**18S**	GTA ACC CGT TGA ACC CCA TT	CCA TCC AAT CGG TAG TAG CG	NR_003278
**Acc**	CTT CCT CCTGAT CAG CAA CTC T	CGT GAG TTT TCC CAA AAT AAG C	NM_133904
**Acox1**	GGGAATTTGGCATCGCAGAC	CATCTCCGTCTGGGCGTAGG	NM_015729
**Cpt1**	GTG TTG GAG GTG ACA GAC TT	CAC TTT CTC TTT CCA CAA GG	NM_013495
**Fas**	TCT GGG CCA ACC TCA TTG GT	GAA GCT GGG GGT CCA TTG TG	NM_007988
**Gpr120**	GCT GCC CCT CTG CAT CTT GT	CTC TTC CGC GAT GCT TTC GT	NM_181748
**Il-1β**	GTC CGA CAG CAC AGA GGC TTT	TGG CTG TGG AGA AGC TGT GG	NM_008361
**iNos**	CCC CTG GAA GTT TCT CTT CAA AGT C	GAT TCT GGA ACA TTC TGT GCT GTC C	NM_010927
**Ir**	CAT CCC GAA AGC GAA GAT CC	GAG TCC TGA TTG CAT GCC TGC AG	NM_001330056
**Irs1**	GCT CTA GTG CTT CCG TGT CC	GTT GCC ACC CCT AGA CAA AA	NM_010570
**Irs2**	GAA GCG GCT AAG TCT CAT GG	GAC GGT GGT GGT AGA GGA AA	NM_001081212
**Lbp**	CTT GGC GTG GTC ACT AAT GT	CTC ACT TGT GCC TTG TCT GG	NM_008489
**Myd88**	CCCTAGGGCAGAGGGGAAGA	ATGCCTGTGTGTGCGAGGAG	NM_010851
**Scd-1**	CCG ATA AAA GGG GGC TGA GG	TGC TGA GAT CGA GCG TGG AC	NM_009127
**Srebp1c**	ACC GGC TAC TGC TGG ACT GC	AGA GCA AGA GGG TGC CAT CG	NM_001313979
**Tlr4**	AGC CAT TGC TGC CAA CAT CA	GCT GCC TCA GCA GGG ACT TC	NM_021297

Acc: acetyl-CoA carboxylase, Acox1: acyl-CoA oxidase 1, Cpt1: carnitine palmitoyltransferase 1, Fas: fatty acid synthase, Gpr120: G protein-coupled receptor 120, Il-1β: interleukin 1 beta, iNos: inducible nitric oxide synthase, Ir: insulin receptor, Irs: insulin receptor substrate, Lbp: lipopolysaccharide binding protein, Myd88: myeloid differentiation primary response 88, Scd-1: stearoyl-CoA desaturase-1, Srebp1c: sterol regulatory element-binding protein-1c, Tlr4: toll-like receptor 4.

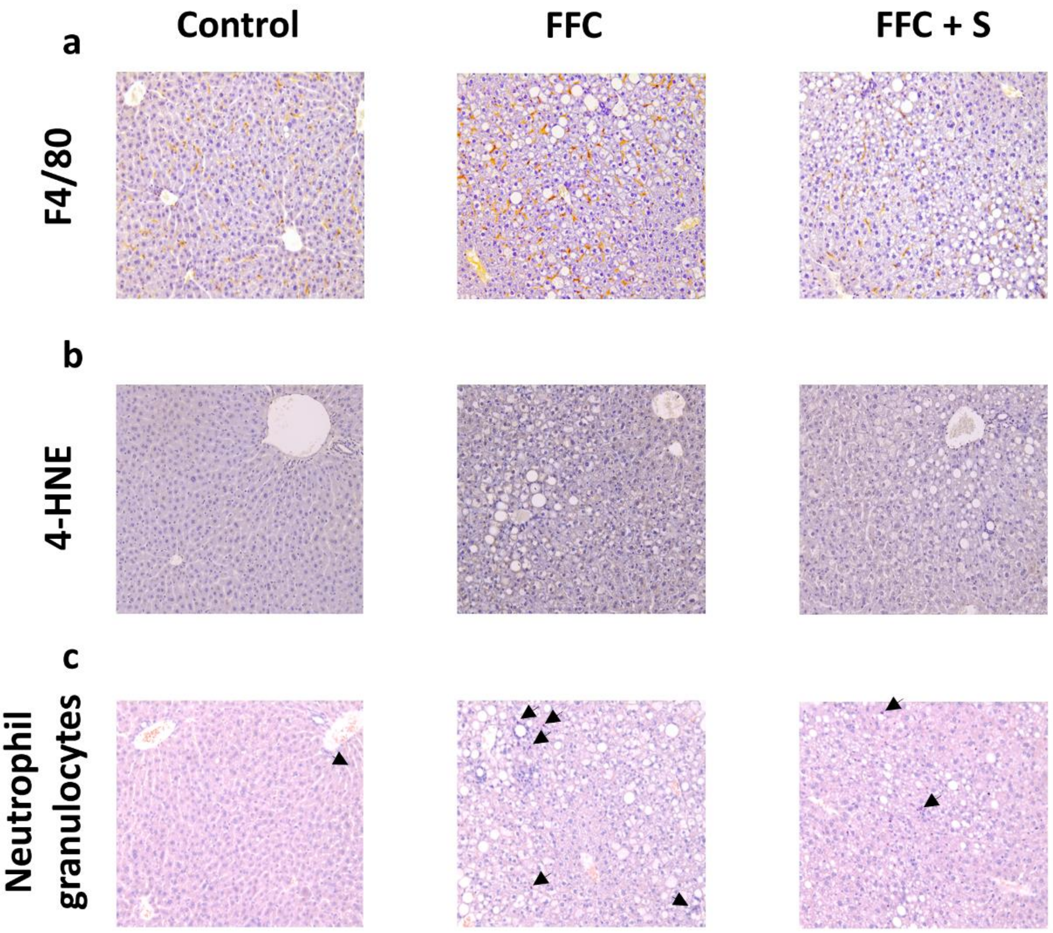

Figure S1: Representative pictures of (a) F4/80 positive cells (200x), (b) 4-hydroxynonenal (4-HNE) protein adduct staining (200x) and neutrophil granulocyte (200x) in liver sections of C-, FFC-, and FFC+S -fed mice. C: control diet, FFC: fat-, fructose- and cholesterol-rich diet, FFC+S, fat-, fructose- and cholesterol-rich diet supplemented with soybean oil. Arrows are indicating stained neutrophil granulocytes.

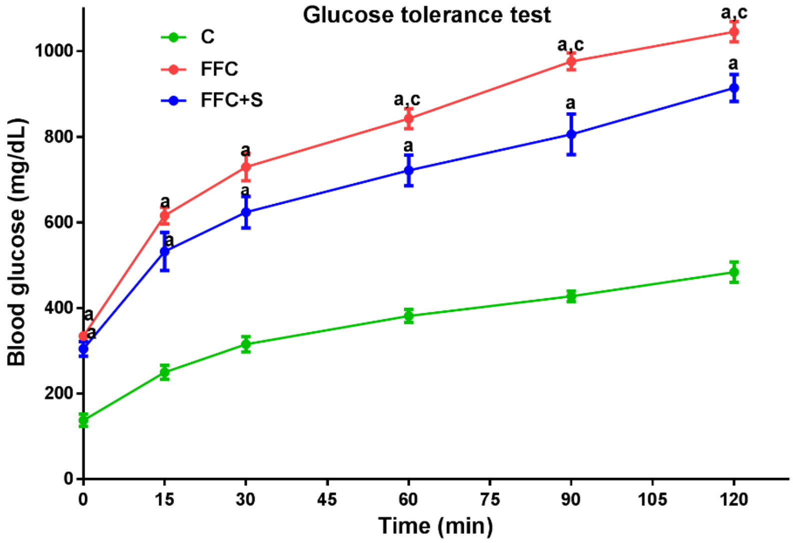

Figure S2: Glucose tolerance test of C-, FFC-, and FFC+S -fed mice. C: control diet, FFC: fat-, fructose- and cholesterol-rich diet, FFC+S, fat-, fructose- and cholesterol-rich diet supplemented with soybean oil. Data are presented as means ± SEM, n = 6-8 mice per group [a]p ≤ 0.05 compared with mice fed the C-diet. [c]p ≤ 0.05 compared with mice fed the FFC+S-diet.
